# Gene expression profiling of the early pathogenesis of wooden breast disease in commercial broiler chickens using RNA-sequencing

**DOI:** 10.1371/journal.pone.0207346

**Published:** 2018-12-05

**Authors:** Michael B. Papah, Erin M. Brannick, Carl J. Schmidt, Behnam Abasht

**Affiliations:** Department of Animal and Food Sciences, University of Delaware, Newark, Delaware, United States of America; Tokat Gaziosmanpasa University, TURKEY

## Abstract

Wooden Breast Disease (WBD), a myopathy in commercial broiler chickens characterized by abnormally firm consistency of the pectoral muscle, impacts the poultry industry negatively due to severe reduction in meat quality traits. To unravel the molecular profile associated with the onset and early development of WBD in broiler chickens, we compared time-series gene expression profiles of Pectoralis (P.) major muscles between unaffected and affected birds from a high-breast-muscle-yield, purebred broiler line. P. major biopsy samples were collected from the cranial and caudal aspects of the muscle belly in birds that were raised up to 7 weeks of age (i.e. market age). Three subsets of biopsy samples comprising 6 unaffected (U) and 10 affected (A) from week 2 (cranial) and 4 (caudal), and 4U and 11A from week 3 (cranial) were processed for RNA-sequencing analysis. Sequence reads generated were processed using a suite of bioinformatics programs producing differentially expressed (DE) genes for each dataset at fold-change (A/U or U/A) >1.3 and False Discovery Ratio (FDR) <0.05 (week 2: 41 genes; week 3: 618 genes and week 4: 39 genes). Functional analysis of DE genes using literature mining, BioDBnet and IPA revealed several biological processes and pathways associated with onset and progress of WBD. Top among them were dysregulation of energy metabolism, response to inflammation, vascular disease and remodeling of extracellular matrix. This study reveals that presence of molecular perturbations involving the vasculature, extracellular matrix and metabolism are pertinent to the onset and early pathogenesis of WBD in commercial meat-type chickens.

## Introduction

The poultry industry is one of the key players contributing to sustainable food sources in the world. Through the utilization of artificial genetic selection, commercial broiler lines with high merit for traits such as increased muscle yield, fast growth rates and feed efficiency have been produced [1–3]. Despite the gains brought about by the use of genetic selection in modern broiler chickens, these chickens have also developed, albeit inadvertently, negative attributes, top among them being increased myodegenerative disorders [4,5]. One such myodegenerative disorder is Wooden Breast Disease (WBD), a myopathy that is uniquely characterized by extreme palpable firmness of the Pectoralis (P.) major muscles in severe cases [6–9]. This disorder has been determined to compromise meat quality traits especially in severe cases [10–12], resulting in significant economic loss to the poultry industry globally.

The gross and histopathological presentation of this disorder in broiler chickens has also been characterized. WBD presents itself palpably as localized foci of firmness starting from the cranial aspects of the P. major muscle, progressing to multifocal and then diffuse distribution [6,7,9]. Grossly, lesions such as outbulging of the lateral forebreasts, muscle hemorrhage and pallor, subcutaneous edema, and in most cases, white striations, are common characteristics of the myopathy. Microscopically, the disorder features lesions of myodegeneration, myonecrosis, inflammatory cell infiltration, characteristic arterial-sparing vasculitis of small caliber venous vessels, and in chronic or severe cases, interstitial fibrosis and myoregeneration [6–9]. Ultrastructural examination undertaken on the affected muscles have revealed degeneration of mitochondria and myofibrillar apparatus, complexed with formation of lipogranulomatous and dense fibrotic tissue straddling the extracellular matrix compartment [7].

Molecular evaluations involving both gene expression and metabolite profiling associated with WBD have been conducted on chickens at market age (week 6 to 8) [13–15]. Indeed, mRNA-gene expression analysis and metabolomics profiles between affected and unaffected chickens suggested presence of altered redox homeostasis and oxidative stress including compromised glucose metabolism as possible biological processes associated with the disease process [13–15]. In addition, *decorin*, a gene responsible for collagen cross-linking was found to be increased in P. major muscle of affected chickens, suggestive of its role in promotion of fibrosis phenotype in advanced WBD [9,16]. While the studies already undertaken on Wooden Breast myopathy provide vital clues regarding the end stage of fulminant Wooden Breast Disease in commercial broiler chickens, the molecular aspects involving the onset and early progression of WBD still remain to be elucidated.

This study, therefore, is aimed at characterizing the molecular profile associated with the onset and progression of WBD in chickens throughout the early growth period (up to 4 weeks of age) before Wooden Breast Disease is typically detectable by palpation. This was achieved by utilizing RNA-sequencing technique on muscle biopsy samples from P. major muscles between affected and unaffected birds belonging to the high-breast-muscle-yield, purebred broiler line. Gene expression analysis, evaluated through Illumina high-throughput sequencing platform used previously [15], has shown great potential in elucidation of the molecular dynamics associated with the disease at market age. This study, therefore, employed the same technique to discern gene expression profiles associated with the disease progression during the typical growing period for broilers. The results from this study are important not only in advancing our understanding of Wooden Breast Disease in modern broiler chickens, but also in efforts directed towards management and prevention of the condition in chickens.

## Materials and methods

### Birds and housing conditions

Chicken husbandry and sample collection methods including experimental procedures used in this study were approved by the Animal Care and Use committee of the College of Agriculture and Natural Resources, University of Delaware (UD), under protocol number 44 07-08-14R. This study utilized muscle biopsy samples collected from a subset of 350 male chickens belonging to the Heritage Breeders line B, a high-breast-muscle-yield, purebred commercial broiler line whose background and genetic features has been described by Fu *et al*. [17,18]. The birds were raised from day-old to a maximum of 49 days post-hatch at UD’s poultry houses following the protocol explained in a related study [7]. Briefly, at placement, all birds were randomly designated to either necropsy or biopsy groups, with the latter being specifically utilized in the current study. The biopsy group initially comprised 100 birds, of which a total of 85 birds were successfully subjected to biopsy regardless of Wooden Breast Disease status at weeks 2 to 5 of age as described below; 13 birds were found dead of unknown causes while 2 were euthanized due to development of leg pathologies during the growth period. At the conclusion of the study (weeks 6 and 7), all birds in both biopsy and necropsy groups were necropsied and samples from the P. major muscle harvested for microscopic analysis to confirm the status of the muscle with respect to the disease.

### Biopsy protocol

The biopsy protocol used in this study involved performing two pectoral microbiopsies [19] on each bird either at weeks 2 and 4 (denoted as B1) or at week 3 and 5 (denoted B2). Thus, collectively, muscle samples collected represented timepoints across the typical broiler growing period. Microbiopsy was accomplished by sampling through the skin into the craniolateral (week 2 or 3) and caudolateral third (week 4 or 5) of the left pectoral muscle respectively. This second sampling location was selected to remove the potential for confounding factors from repeated sampling at or near the original biopsy site (i.e. resolving hemorrhage, inflammation, or fibrosis at the healing biopsy site). Prior to biopsy, the selected site was plucked of feathers if present, anaesthetized locally via topical application of lidocaine cream (2-4ml) covering the entire cranial or caudal portion of the pectoral muscle, and aseptically prepared with betadine scrub using cotton wool balls. Each biopsy specimen was collected with a sterile Bard Max-Core disposable biopsy instrument with a 16-gauge biopsy needle and 22mm sample notch directed to the P. major muscle at ~ 45 degrees angle to the keel. This orientation facilitated sampling several muscle fibers over the length of the biopsy needle sampling notch. Once collected, the skin and any visible fat tissue was removed from the biopsy core, before the remaining muscle specimen was flash-frozen individually in liquid nitrogen and stored at -80° C for RNA-seq analysis. Only the left P. major muscle was biopsied, while the right was spared for internal control to be sampled for necropsy at week 6 or 7 for histological diagnosis of Wooden Breast Disease status. Following biopsy, bleeding was controlled by application of direct pressure at the site which was then cleaned and covered with iodine cream prior to release of birds. In addition, to allow for a close observation of the chickens and prevent wound contamination immediately after the biopsy procedure, biopsied birds were kept separate from the rest of the flock in clean area covered by a brown Manilla paper for 2 hours prior release. All biopsied birds were then allowed to grow until the end of the experimental period (week 6 and 7) where they were necropsied and evaluated both at gross and microscopic levels for status of Wooden Breast Disease.

### Selection of biopsy samples for RNA-seq analysis

Identification of specific samples from the 85 stored muscle biopsy samples to be processed for RNA-seq analysis was accomplished retrospectively using several parameters previously used for scoring WBD [7]. Briefly, the parameters comprised: (1) Evaluation of gross presentation of the pectoral muscle including presence and distribution of lesions such as hemorrhage, subcutaneous edema, changes in muscle color or pallor were used. At this stage, the WBD scores of the biopsied chickens at necropsy (denoted “gross WBD”), score range (0 unaffected, to 4/5 markedly affected) were used. In addition, live bodyweights of the birds prior to necropsy were considered; those whose bodyweights were lower than 3 standard deviations from the average were excluded from the study. (2) Analysis of microscopic presentation and respective lesion scores of Wooden Breast after necropsy at week 6 and 7, score range (0 unaffected, to 4/5 markedly affected) [7]. From all these assessments, biopsied birds were able to be defined as either “affected” or “unaffected” for Wooden Breast Disease at market age (week 6 and 7). Parameters and scores used to define and categorize affected and unaffected birds in this study are summarized under (S1 Table). Therefore, with the knowledge of the disease states of the biopsied birds at market age, the biopsy muscle samples previously harvested and stored were used to trace back on the early molecular presentation of WBD states at their respective time points i.e. weeks 2, 3 and 4 in the selected birds. A sample size comprising 6 unaffected and 10 affected muscle samples from both weeks 2 and 4 were identified for RNA-seq analysis, using paired samples from the same individual birds at each time point (i.e. 32 total samples from 16 individual birds at 2 time points). Likewise, 4 unaffected and 12 affected (6 moderately and 6 severely affected) samples were identified from weeks 3 and 5 categories for RNA-seq evaluations. To decipher genes associated with early pathogenesis of WBD, the key focus of this study, only sample categories from weeks 2, 3 and 4 were processed for RNA sequencing (Table 1). It should be noted that the number of samples in A and U groups in week 2 and 4 differ from those of week 3. This difference in sample size resulted from the low number of unaffected samples identified for week 3 compared to those for week 2 and 4, following the application of the multi-parametric method indicated above.

**Table 1:** Number of broiler chicken superficial pectoral muscle samples used for RNA-sequencing analysis by bird age.

Bird Age (Week)	Biopsy site	Unaffected	Affected
2	Cranial	6	10
3	Cranial	4	12
4	Caudal	6	10

### RNA extraction

Total RNA was extracted from all selected samples using mirVana^TM^ miRNA Isolation Kit (Thermo Fisher Scientific) following manufacturer’s protocol. The isolated RNA was stored at -80° C until the next stage of cDNA library preparation. Concentration and quality of the RNA samples were measured using NanoDrop 1000 (Thermo Fisher scientific) and Fragment Analyzer at Delaware Institute of Biotechnology (DBI), Newark, DE. For all RNA samples, the RNA quality integrity number (QIN) from fragment analysis was above 6, which was acceptable for cDNA library preparations.

### RNA-seq protocol

Library preparation of cDNA from the samples was performed using the TruSeq Stranded mRNA Sample Prep Kit for low sample protocol (Illumina) following manufacturer’s instructions. During processing, each sample of cDNA was barcoded with a unique index to allow pooling at the final stage of the cDNA library preparations. The constructed cDNA libraries were assessed for concentration and quality using Nanodrop 1000 (UD) and Fragment analyzer (DBI) respectively following the manufacturer’s protocol. All the cDNA libraries passed the quality test and were subsequently normalized to 10 nM/μl using Tris buffer (Tris-Cl 10 mM, 0.1% Tween 20, pH 8.5). From the normalized samples, 10μl of each sample (total of 16 samples per week) was pooled into one tube for each week (total of 3 weeks) of the experiment and subsequently submitted to DBI for paired-end 2x75-nucleotide sequencing using Illumina HiSeq 2500 sequencer. The resulting sequence reads were checked for quality using FastQC program (v0.11.3) [20]. All the sequences passed the quality check, (namely basic statistics, per base sequence quality, per sequence GC content and per base K content), and were subsequently used for mapping to the chicken reference genome (Ensembl Gallgall5.0 May 2017 and gene transfer format (GTF), file release 88) using HISAT2 v2.0.5, software package [21]. Further, Cuffdiff v2.2.1 [22] was used for identification of differentially expressed (DE) genes between affected and unaffected groups. Genes were considered to be statistically significant, and hence, differentially expressed when the test statistic for the mean difference of Fragments Per Kilobase of transcript per Million mapped reads (FPKM) between unaffected and affected groups yielded a q-value or FDR adjusted p-value of <0.05 and fold-change >1.3.

Additionally, one severely affected sample (sample ID: 1683_wk3_Sev2) from week 3 dataset was detected as misclassified in the affected group and therefore, the entire set was re-analyzed in Cuffdiff to identify DE genes without this sample (4 unaffected and 11 affected sample). Further, an analysis consisting of the 6 moderately and 5 severely affected samples from week 3 dataset was performed and DE genes were analyzed to determine whether gene expression profiles at week 3 of the affected group of chickens (moderate and severe) were consistent with their respective phenotypic presentations at week 6 and 7. To ensure that all DE genes used in this study were free from potential skin contamination inadvertently obtained during the biopsy sampling procedure, a list of skin-derived DE genes was generated from week 3 samples (S1 File). Any gene with expression level of log2FC ≥1 from this skin-derived gene list was considered as a potential skin contaminant. Therefore, DE gene-sets obtained from all 3 time points were first scrutinized for potential skin contamination by comparing with the skin-derived gene list, and any contaminant subsequently removed if present. The resulting DE genes across all 3 timepoints were then used for downstream functional analysis.

### Analysis of DE genes

Functional analysis of DE genes from week 2 and 4 was accomplished using BioDBnet software [23] and literature mining. Functional analysis of DE genes from week 3 dataset was accomplished using Ingenuity Pathway Analysis (IPA) program. Briefly, all DE genes were submitted to IPA for functional annotation and identification of canonical pathways and upstream regulators.

## Results

### Differentially expressed genes

Sequencing of cDNA libraries produced an average of 34 million reads per sample in week 2; 33 million reads in week 3 and 39 million reads in week 4, all counted as paired-end. Raw sequence reads in each week were processed and analyzed producing DE genes between affected and unaffected categories across the 3-week experimental period. In this case, there were 41 and 39 DE genes in weeks 2 and 4 respectively, with directionality of the genes shown in (Table 2). Week 3 dataset comprising 4 unaffected vs 11 affected yielded 618 DE genes (Table 2). Of the 618 DE genes, 504 genes were identified as mapped by IPA software and were therefore used for functional analysis downstream. Moreover, within the affected group in week 3, there were 30 DE genes between 6 moderately and 5 severely affected muscle samples (Table 2).

**Table 2:** Differentially expressed (DE) genes from week 2, 3 and 4 sample sets comparing wooden breast disease unaffected (U-AFF) to affected (AFF) or moderately-affected (M-AFF) to severely-affected (S-AFF) broiler chicken pectoral muscle samples.

	Pectoral major muscle sample			Number of DE genes^2^		
Week^1^	Location	Condition 1 (n)	Condition 2 (n)	Total	Up	Down
2	Cranial	U-AFF (6)	AFF (10)	41	18	23
3	Cranial	U-AFF (4)	AFF (11)	618	251	367
3	Cranial	M-AFF (6)	S-AFF (5)	30	17	13
4	Caudal	U-AFF (6)	AFF (10)	39	18	21

^1^ Week 2 and week 4 samples were taken from the same birds. Moderately (n = 6) and severely (n = 5) affected samples at week 3 were combined as one condition (n = 11 affected) to be compared with the other condition (unaffected).

^2^Upregulated (Up) and downregulated (Down) genes in the affected or severely affected group.

### Overlapping genes

**Fig 1:**
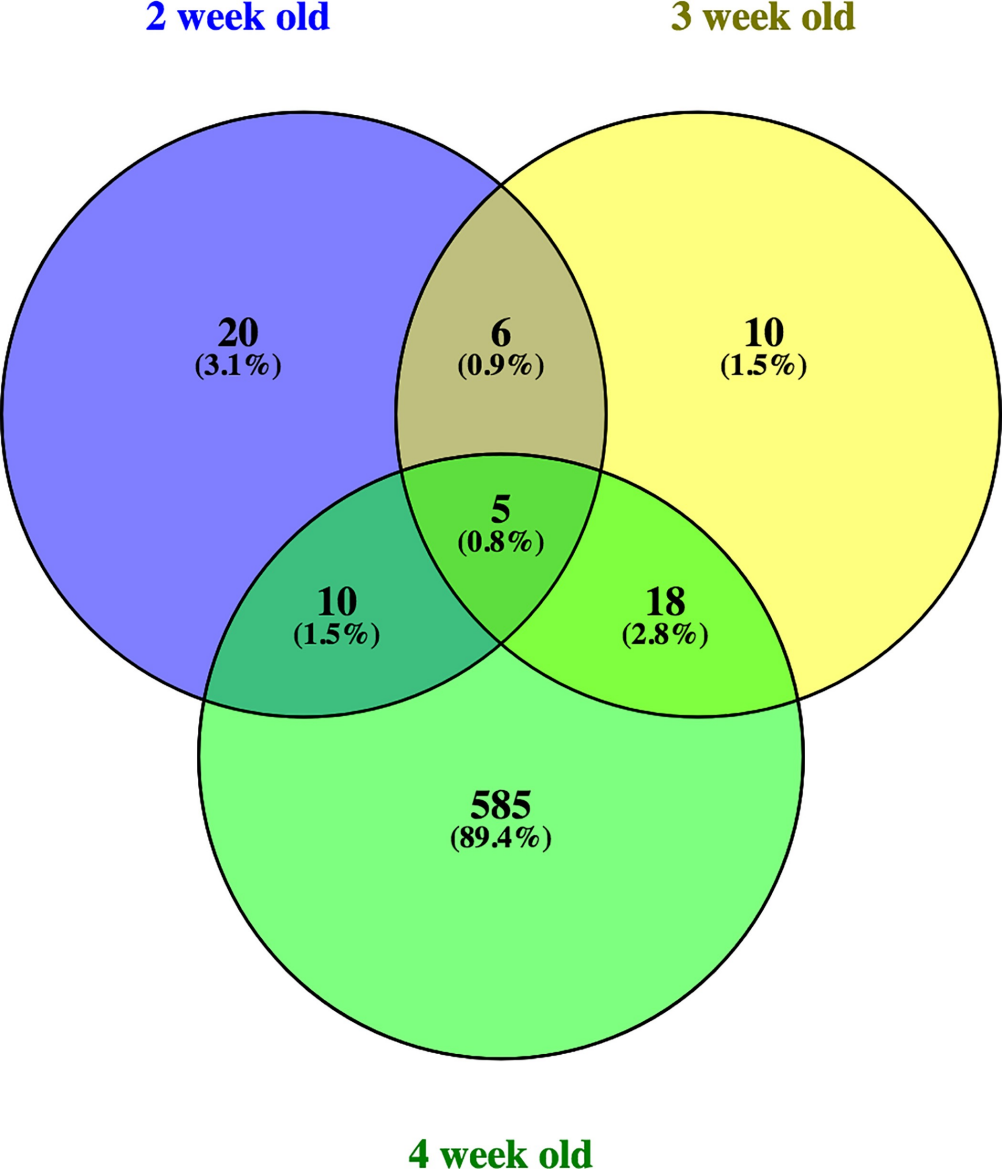
Overlapping differentially expressed genes across 3-week period. Differentially expressed genes between Wooden Breast Disease affected and unaffected pectoral muscle showing overlap across broiler chickens at 2, 3 and 4 weeks of age. Only 5 DE genes were found in common at all time points.

Comparison of DE genes between week 2 and 4, whose biopsy samples were extracted from the same birds revealed 11 overlapping genes as shown in (Table 3). Of all 11 overlapping genes, 5 genes were upregulated, and the same number of genes were also downregulated, while only one gene (*ENSGALG00000044239*) showed opposing directionality (Table 3) in WBD affected chickens. Among the top upregulated genes in both weeks included *fibromodulin* (*FMOD*), *crumbs 2* or *cell polarity complex component* (*CRB2*) and *collagen type XII alpha 1 chain* (*COL12A1*). Conversely, the top downregulated genes for week 2 and 4 included *sidekick cell adhesion molecule 1* (*SDK1*), *ENSGALG00000019175*, *kyphoscoliosis peptidase* (*KY*), and *stratifin* (*SFN*). Similarly, the overlap of DE genes in all 3 weeks showed only 5 genes, namely *CRB2*, *COL12A1*, *KY*, *ENSGALG00000030512* and *SDK1* (Table 4). The number of overlapping genes between week 2 and 3 was 15, where 9 genes showed consistency of expression pattern in both weeks while 6 genes showed opposing expression pattern (Fig 1 and S2 Table). On the other hand, 26 DE genes were found to be unique in week 2 while 603 genes were unique in week 3 (S2 Table). The gene overlap between week 3 and week 4 was 23 (Fig 1).

**Table 3:** DE genes between wooden breast disease affected and unaffected pectoral muscle samples showing overlap between age groups at weeks 2 and 4.

GENE ID		Gene symbol	Gene name	Log2FC Week2	Log2FC Week4
*ENSGALG00000034067*		*FMOD*	*Fibromodulin*	↑0.66	↑1.25
*ENSGALG00000001169*		*CRB2*	*Crumbs 2*,	↑1.45	↑1.12
*ENSGALG00000015908*		*COL12A1*	*Collagen type XII alpha 1 chain*	↑0.77	↑1.08
*ENSGALG00000041266*		*CNN1*	*Calponin 1*	↑0.68	↑1.01
*ENSGALG00000005024*		*EFCC1*	*EF-hand and coiled-coil domain containing 1*	↑1.02	↑0.87
*ENSGALG00000044975*		*KY*	*kyphoscoliosis peptidase*	↓0.86	↓0.75
*ENSGALG00000004420*		*SDK1*	*Sidekick cell adhesion molecule 1*	↓1.09	↓0.79
*ENSGALG00000044239*		*-*	*-*	↑0.80	↓0.87
*ENSGALG00000030512*		*-*	*-*	↓0.86	↓1.09
*ENSGALG00000019175*		*LOC418544*	*Cystathionine beta-synthase-like*	↓1.03	↓1.43
*ENSGALG00000028115*		*SFN*	*Stratifin*	↓0.81	↓2.41

Log2FC of a gene is determined by log2 (FC = affected _FPKM_/unaffected _FPKM_). The dashes (-) indicate gene symbols and names are still unknown.

↑ indicates upregulation in affected group

↓ indicates downregulation in affected group

Log2FC values are negative for downregulated genes

**Table 4:** DE genes between wooden breast disease affected and unaffected pectoral muscle samples showing overlap among age groups at weeks 2, 3 and 4 of age.

Gene ID	Gene symbol	Gene name	Log2FC Wk2	Log2FC Wk3	Log2FC Wk4
*ENSGALG00000004420*	*SDK1*	*Sidekick cell adhesion molecule 1*	↓1.09	↓0.51	↓0.79
*ENSGALG00000044975*	*KY*	*Kyphoscoliosis peptidase*	↓0.86	↓0.73	↓0.75
*ENSGALG00000001169*	*CRB2*	*Crumbs 2*, *cell polarity complex component*	↑1.45	↓0.56	↑1.12
*ENSGALG00000030512*	*-*	*-*	↓0.86	↓0.93	↓1.09
*ENSGALG00000015908*	*COL12A1*	*Collagen type XII alpha 1 chain*	↑0.77	↑1.05	↑1.08

Log2FC of a gene is determined by log2 (FC = affected _FPKM_/unaffected _FPKM_). The dashes (-) indicate gene symbols and names are still unknown.

↑ indicates upregulation in affected group

↓ indicates down regulation in affected group

Log2FC values are negative for downregulated genes

### Functional analysis of differentially expressed genes from week 2 and 4 post-hatch

In week 2, the main biological terms whose genes were upregulated in the pectoral muscles of affected chickens included skeletal muscle differentiation, ECM receptor interaction stress/oxidative stress and response to inflammation. Conversely, the biological terms whose genes were downregulated in pectoral muscles of affected chickens included cell adhesion and metabolic pathways (Table 5). In week 4, the biological pathways whose genes were upregulated in affected birds included cell adhesion while those that were downregulated included ATP-binding and metabolic pathways (Table 6).

**Table 5:** Top pathways/biological terms from DE genes upregulated or downregulated in wooden breast disease affected pectoral muscle as compared to unaffected pectoral muscle in broiler chickens 2 weeks of age.

Biological term/Pathway	Gene symbol	Gene Full Name	RNA-seq-Log2FC
Cell adhesion	*COL12A1*	*Collagen type XII alpha 1 chain*	↑0.77
	*SFN*	*Stratifin*	↓0.81
	*SDK1*	*Sidekick cell adhesion molecule 1*	↓1.09
	*GJD2*	*Gap junction protein delta 2*	↓1.08
Metabolic pathways	*CHAC1*	*ChaC glutathione specific gamma-glutamylcyclotransferase 1*	↑0.84
	*TMEM68*	*Transmembrane protein 68*	↓0.82
	*PDP1*	*Pyruvate dehydrogenase phosphatase catalytic subunit 1*	↓0.54
	*G0S2*	*G0/G1 switch 2*	↓0.86
	*LOC418544*	*Cystathionine beta-synthase-like*	↓1.03
Stress/Oxidative stress	*FMOD*	*Fibromodulin*	↑0.66
	*ATF3*	*Activating transcription factor 3*	↑0.82
	*ANKRD1*	*Ankyrin repeat domain 1*	↑0.87
ECM-receptor interaction	*FMOD*	*Fibromodulin*	↑0.66
	*ATF3*	*Activating transcription factor 3*	↑0.82
	*ANKRD1*	*Ankyrin repeat domain 1*	↑0.87
Response to inflammation	*ATF3*	*Activating transcription factor 3*	↑0.82
	*ANKRD1*	*Ankyrin repeat domain 1*	↑0.87
	*G0S2*	*G0/G1 switch 2*	↓0.86
Skeletal muscle differentiation	*ASB2*	*Ankyrin repeat and SOCS box containing 2*	↑0.56
	*ATF3*	*Activating transcription factor 3*	↑0.82
	*ANKRD1*	*Ankyrin repeat domain 1*	↑0.87
Transport	*AQP4*	*Aquaporin 4*	↑0.80
	*ALS2*	*Amyotrophic lateral sclerosis 2 (juvenile)*	↑0.58
	*SLC20A1*	*Solute carrier family 20 member 1*	↓0.75

↑ indicates upregulation in affected group

↓ indicates down regulation in affected group

Log2FC values are negative for downregulated genes

**Table 6:** Top pathways/biological terms from DE genes between wooden breast disease affected compared to unaffected pectoral muscle in broiler chickens at 4 weeks of age.

Biological term/Pathway	Gene symbol	Gene Full Name	RNA-seq-Log2FC
Cell adhesion	*ITGB2*	*Integrin subunit beta 2*	↑0.87
	*TNC*	*Tenascin C*	↑0.82
	*SPP1*	*Secreted phosphoprotein 1*	↑1.75
	*COL12A1*	*Collagen type XII alpha 1 chain*	↑1.08
	*CNTN1*	*Contactin 1*	↓0.92
ATP binding	*KIF21A*	*Kinesin family member 21A*	↓0.55
	*ASNS*	*Asparagine synthetase (glutamine-hydrolyzing)*	↓0.81
	*ABCA12*	*ATP binding cassette subfamily A member 12*	↓3.12
Metabolic pathways	*SLC16A9*	*Solute carrier family 16 member 9*	↓0.86
	*ABCA12*	*ATP binding cassette subfamily A member 12*	↓3.12

↑ indicates upregulation in affected group

↓ indicates down regulation in affected group

Log2FC values are negative for downregulated genes

### Functional analysis of differentially expressed genes from week 3 post-hatch

Differentially expressed genes whose gene symbols were identified and mapped numbering 504 of the total 618 were used to perform functional analysis on IPA program. Several functional categories associated with the DE genes as revealed by the IPA program included top canonical pathways, diseases and biological functions, as well as top upstream regulators of the DE genes.

### Top canonical pathways

**Fig 2:**
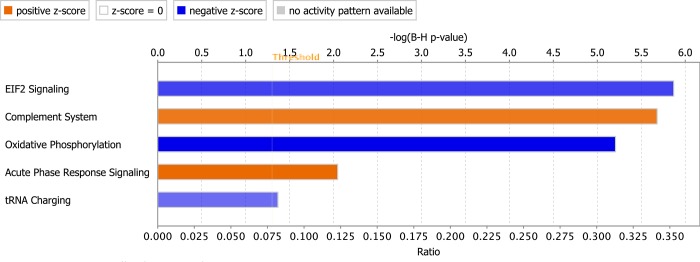
Top canonical pathways (Z-scores >1.8) as predicted by IPA for pectoral muscles at week 3 of age. Activated pathways are represented by orange-colored bars (color shade increasing with strength of activation) while inhibited pathways are shown by blue-colored bars (color shade increasing with the strength of inhibition).

Evaluation of DE genes at week 3 for top canonical pathways (Z-scores >1.8) with Benjamin-Hochberg (B-H) multiple testing correction P-value ≤ 0.05 revealed pathways predicted to be activated and inhibited in affected chickens. Pathways predicted to be activated included complement system (Z-score = 1.89) and acute-phase response signaling (Z-score = 2.12). Conversely, the pathways predicted to be inhibited in the pectoral muscles of affected chickens included EIF2 signaling pathway (Z-score -2.89), oxidative phosphorylation (Z-score -3.87) and tRNA charging (Z-score -2.24) (Fig 2).

### Diseases and biological functions

**Fig 3:**
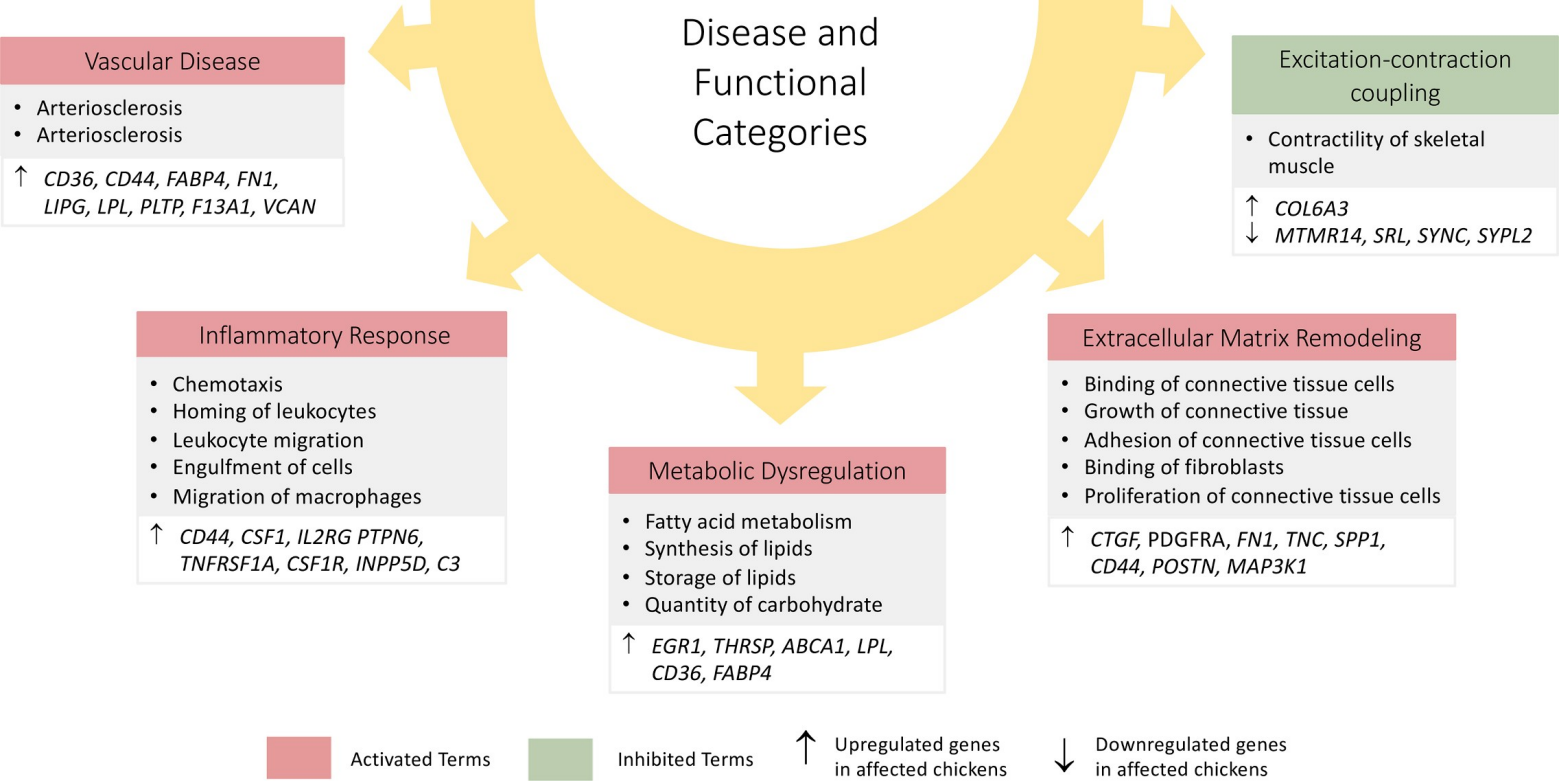
Disease and functional characterization of DE genes from the pectoral muscles at week 3 of age as predicted by IPA. Activated terms have (Z-scores ≥ 2) while inhibited terms have (Z-scores ≤ -2). Each biological term has cluster annotation(s) associated with it. In addition, the main genes associated with the cluster annotation(s) including their expression states with respect to affected chickens are shown.

Analysis of DE genes for disease and biological functions in IPA revealed a number of biological processes as depicted by significant (Z-scores ≥ 2) for those predicted to be activated or (Z-scores ≤ -2) for those predicted to be inhibited in pectoral muscles of affected chickens. For convenience purposes, the biological and disease processes identified are grouped into the following disease and functional categories: vascular disease; inflammatory response; metabolic dysregulation; extracellular matrix (ECM) remodeling and excitation-contraction coupling (Fig 3). Specific molecules (DE genes from the current dataset) and biological annotations associated with the above disease and functional categories are found in (S3 Table).

#### Vascular disease

Vascular disease process was predicted to be activated (Z-score of >2) in the breast muscles of affected chickens based on the identification of genes enriched for atherosclerosis and arteriosclerosis processes. The genes associated with these processes included *CD36 molecule or fatty acid translocase (CD36)*, *CD44*, *fatty acid binding protein 4 (FABP4)*, *FN1*, *lipase G* (*LIPG*), *lipoprotein lipase* (*LPL*), *phospholipid transfer protein* (*PLTP*) and *versican* (*VCAN*) (Fig 3).

#### Inflammatory responses

Analysis of DE genes from week 3 samples revealed activation of several biological processes under inflammatory response cluster category in affected birds (Fig 3). These processes included, chemotaxis of immune cells, homing of leukocytes, leukocyte migration, engulfment of cells and migration of phagocytes (Fig 3).

#### Metabolic dysregulation

Metabolic dysregulation was predicted to be increased in the pectoral muscles of the affected chickens as depicted by 4 cluster annotations, namely fatty acid metabolism, synthesis of lipid, storage of lipid and quantity of carbohydrates. The 4 clusters were enriched with several genes related to metabolism such as *early growth response 1* (*EGR1*), *thyroid hormone responsive* (*THRSP*), *ATP binding cassette subfamily A member 1* (*ABCA1*), *LPL*, *CD36* and *FABP4* (Fig 3).

#### Extracellular matrix remodeling

Extracellular matrix (ECM) remodeling was predicted to be activated based on the significant Z-scores (≥2) of each of the 5 related clusters. The clusters included binding of connective tissue cells, growth of connective tissue, adhesion of connective tissue cells, binding of fibroblasts and proliferation of connective tissue cells (Fig 3). Several enriched genes such as *secreted phosphoprotein 1* (*SPP1*), *fibronectin* (*FN1*), *CD44 molecule (CD44*), *periostin* (*POSTN*), *connective tissue growth factor* (*CTGF*) and *mitogen-activated protein kinase kinase kinase 1* (*MAP3K1*) were associated with all the cluster annotations (Fig 3).

#### Excitation-contraction coupling

Functional analysis of DE genes at week 3 of age using IPA revealed inhibition of contractility in affected muscles (Z score < -2) (S3 Table). Several genes related to this pathway were downregulated in affected chickens including *myotubularin related protein 14* (*MTMR14*), *sarcalumenin* (*SRL*), *syncoilin*, *intermediate filament protein* (*SYNC*) and *synaptophysin like 2* (*SYPL2*) (Fig 3).

### Upstream regulators

Analysis of DE genes from P. major muscles at week 3 of age for upstream regulators of expressed genes and related functions in IPA revealed several significant biological molecules predicted to be activated (Z-scores ≥2) (S4 Table**)** or inhibited (Z-scores ≤ -2) (S5 Table**).** The upstream regulators identified based on the DE gene profile from week 3 belonged to a diverse group of biological molecules including enzymes, transcription regulators, transmembrane receptors, translation regulators, growth factors, cytokines, and endogenous chemicals (S4 and S5 Tables). Of all the upstream regulators detected, 8 were part of the DE gene list submitted to IPA where 7 were upregulated and also predicted to be activated in affected chickens, while 1 gene namely, *VCAN*, which was upregulated in affected chickens, displayed opposing prediction state (Table 7). The 7 upstream regulators included *CCAAT/enhancer binding protein alpha* (*CEBPA*), *CD44*, *complement C3* (*C3*), *mitogen-activated protein kinase kinase kinase 1* (*MAP3K1*), *Spi-1 proto-oncogene* (*SPI1)*, *colony stimulating factor 1* (*CSF1*) and *FN1* (Table 7).

**Table 7:** Differentially-expressed (DE) upstream regulators and their respective target genes in the gene set of wooden breast disease affected and unaffected pectoral muscle from broiler chickens at 3 weeks of age.

Upstream Regulator symbol	Upstream regulator name	Expression Log2FC	Molecule Type	Target molecules in dataset
CEBPA*	*CCAAT/enhancer binding protein alpha*	↑1.37	transcription regulator	*CSF1*, *CSF1R*, *FABP4*, *LPL*, *PLIN1*, *C3*, *C3AR1*
CD44*	*CD44 molecule*	↑1.24	other	*CD36*, *CD44*, *CLU*, *FN1*, *SDC4*, *SPP1*
C3*	*Complement C3*	↑1.08	peptidase	*C1QA*, *C3AR1*, *CSF1*, *FN1*, *UCP3*
MAP3K1*	*Mitogen-activated protein kinase kinase kinase 1*	↑0.88	kinase	*EGR1*, *FOS*, *NOV*, *PTGS2*, *TNC*
SPI1*	*Spi-1 proto-oncogene*	↑0.87	transcription regulator	*CEBPA*, *CSF1*, *CSF1R*, *KLF4*, *PTGS2*
CSF1*	*Colony stimulating factor 1*	↑0.72	cytokine	*CEBPA*, *CSF1R*, *EGR1*, *FN1*, *THBS1*
FN1*	*Fibronectin 1*	↑0.59	enzyme	*ACP5*, *FN1*, *PDGFRA*, *SPP1*, *THBS1*
*VCAN†*	*Versican*	↑0.80	other	*C3*, *CIDEA*, *CLU*, *COMP*, *DAG1*

The symbols show prediction activation states of the upstream regulators as provided by IPA

where asterisk (*) indicates activated

while obelisk (†) indicates inhibited prediction states.

Notice that one upstream regulator namely *Versican* (*VCAN*) shows contrasting prediction state with its expression pattern. The prediction states of the rest of the upstream regulators are consistent with their expression profiles.

↑ indicates upregulation of the gene in affected group

## Discussion

This study examined the gene expression profile associated with the onset and progression of Wooden Breast Disease in modern broiler chickens over the early growth period (week 2 to 4). While knowledge on the molecular profile of WBD at market age (week 6 to 8 post-hatch) is available [13,15], it is not sufficient to understand the early pathogenesis of the disease due to the advanced state of inflammation and fibrosis in affected birds of that age. Therefore, by evaluating the pectoral muscle biopsy samples harvested from affected and unaffected 2 to 4-week-old broiler chickens using RNA-seq analysis, it was possible to discern pertinent molecular features highly likely to be associated with the early pathogenesis of the Wooden Breast syndrome. It should be noted that the selection of biopsy muscle samples in this study was correlated to gross and histologic WBD status of the same birds at market age (week 6 and 7).

The current study showed relatively low number of differentially expressed genes between affected and unaffected birds from week 2 with 41 DE genes (cranial pectoral region) and week 4 with 39 DE genes (caudal pectoral region). Therefore, the disease may be thought to assume a spatiotemporal distribution whereby the caudal pectoral muscle at week 4 of the pectoral regions exhibits a similar stage of the disease process as the cranial muscle at week 2. This observation is further supported by similar directionality of the overlapping genes (n = 10/11) between week 2 and week 4 observed in the current study.

### Functional analysis of DE genes of biopsy muscles at weeks 2 and 4 post-hatch

Functional analysis of DE genes from week 2 indicated enrichments for biological processes such as oxidative stress, ECM receptor interaction, skeletal muscle differentiation and response to inflammation. The observation of enrichment in skeletal muscle differentiation in particular suggests an earlier occurrence of myoregeneration than previously encountered (3 weeks of age), likely indicating the molecular activity preceding microscopically discernible evidence of regenerative myotubes [7]. Even though the gene sets supporting the biological processes are fewer, these genes are indicative of their potential significance to the disease. In addition, a previous study reported fewer lesions as well as lower incidence of the lesions in the early stages of the disease such as localized phlebitis at week 1 followed by focal myositis, degeneration and phlebitis at week 2 [7,8], which further corroborates the low number of DE genes at week 2 in this study, and correlates with the early phases of the myopathy. Further, the finding of inflammation and oxidative stress in muscles of affected chickens at week 2 is a confirmation of the early subclinical onset of the myopathy which then increases in scope, clinical significance, and lesion severity towards market age (week 7) as demonstrated by the gene expression profile [13,15].

Functional analysis of gene expression profile at week 4 (caudal pectoral muscles) showed enrichments for cell adhesion in chickens with WBD. On the other hand, affected chickens showed decreased functions in ATP binding and metabolic pathways. These processes are in tandem with features of the early phase of the Wooden Breast myopathy. For example, increase in cell adhesion activity, which serve to prime inflammatory response through interaction of endothelial cells and leukocytes [24], may be associated with the initiation of the vasculopathy (phlebitis) reported in the early stages of Wooden Breast [7].

### Transcriptomic analysis of biopsy muscles at week 3 post-hatch

Analysis of cranial pectoral muscle biopsy samples between unaffected and affected chickens from week 3 showed higher numbers of DE genes (n = 618) compared to both week 2 (n = 41) and week 4 (n = 39) muscle biopsy samples. This observation supports the findings of histological analysis of the pectoral muscles on the same group of birds that reported advancement in pathology of WBD and increase in incidence of muscle lesions associated with WBD for birds at week 3 compared to those at week 2 post-hatch [7]. Based on these observations, it is evident that increasing scope and severity of pectoral muscle pathology as a result of WBD is also manifested by divergence of the transcriptome profile between unaffected and affected phenotypes over time with advancing disease. Indeed, in the current study, a comparison of DE genes in the cranial pectoral region between week 2 and 3 revealed 26 unique genes (13 upregulated and 13 downregulated) at week 2 compared to 603 unique genes (243 upregulated and 358 downregulated) at week 3, with only 15 genes being common at both weeks (see S2 Table). This finding also suggests that stage-wise progress of Wooden Breast in chickens, parallels increasing recruitment of gene expression associated with the myopathy. The low number of overlapping DE genes (n = 5) among the 3-week-experimental period further confirms that the pectoral muscle transcriptome is not necessarily similar for a given severity classification across different stages of the myopathy.

Evaluation of the differential transcriptome between the moderately and severely affected biopsy muscle samples at week 3 of age revealed low number of DE genes (n = 30) as opposed to the comparison between unaffected and the combined “affected” bird category (comprising moderate and severely affected birds) which yielded (n = 618). Even though there were distinct phenotypic and histologic differences between the moderate and severely affected chickens that necessitated their grouping at market age (week 6 and 7), the same birds did not exhibit much variation of the breast muscle transcriptome at 3 weeks of age. This is an indication that the moderate and severely affected chickens were at the same stage of the myopathy at week 3. However, in subsequent weeks, differential rates of development of the disease among birds appeared to have occurred resulting in discernable differences in severity of the disease at market age (week 6 and 7). This phenomenon shows an apparent existence of time-dependent, multiphasic pattern involving the pathogenesis of WBD in modern broiler chickens. Based on this finding, a time frame at or before 3 weeks of age could be targeted for mitigation strategies for the myopathy.

### Functional analysis of DE genes of P. major muscles at week 3

Analysis of top canonical pathways by IPA predicted inhibition of the eukaryotic initiation factor 2 (EIF2) signaling pathway as well as tRNA charging, suggesting a potential occurrence of translation attenuation in affected chickens. The fast-growth rate and increased breast-muscle-weight phenotype frequently exhibited by modern broiler chickens suggests that protein synthesis is also accelerated in comparison with unselected chickens. Consequently, it may be argued that the unprecedented increase in protein synthesis potentially puts more strain on the endoplasmic reticulum (ER)/sarcoplasmic reticulum (SR) to optimize protein folding. Eukaryotes generally monitor protein synthesis and folding in the ER to maintain homeostasis especially for proteins that are processed in the ER [25]. Accordingly, eukaryotic cells activate unfolded protein response (UPR) in the event of increased accumulation of unfolded proteins within the ER, frequently occasioned by elevated protein synthesis [25]. One way in which UPR is activated is through phosphorylation of the eukaryotic translation initiation factor 2 alpha (eIF2a), resulting in translation attenuation of mRNA, thereby allowing the unfolded proteins to be cleared by the cell and subsequent restoration of ER homeostasis [26]. While we did not examine phosphorylation events of any molecules in our current study, the downregulation of several genes involved in the EIF2 signaling pathway may serve to attenuate translation of mRNAs, whose effect would be more or less similar as those of phosphorylation of eIF2a. Based on this observation, the downregulation of genes involved in the EIF2 signaling pathway in affected chickens, may be a regulatory response employed by the P. major muscles following increased protein synthesis that is beyond the capacity of the ER/SR to handle. Therefore, attenuation of translation at the gene expression level may aid in preventing the potential buildup of the deleterious unfolded proteins in ER/SR of the muscles of affected chicken. It is also worth noting that several genes involved in the ubiquitin-proteasome pathways were downregulated in affected chickens. These genes include *ubiquitin specific peptidase 2* (*USP2*), *valosin containing protein* (*VCP*), *proteasome 26S subunit*, *ATPase 5* (*PSMC5*), *ubiquitin fusion degradation 1 like* (*UFD1L*) and *proteasome subunit beta 4* (*PSMB4*) (see S2 Table). The downregulation of these genes suggests a potential reduction of ubiquitin-proteasome activity in affected chickens, which may lead to a buildup of damaged, misfolded or dysfunctional proteins possibly triggering the inhibition of the EIF2 signaling pathway. The decreased ubiquitin-proteasome activity could also be reflective of the increasing levels of oxidative stress [27] in the pectoral muscles of affected chickens that was initially reported in chickens at market age [14,15].

Inhibition of oxidative phosphorylation suggests a compromised energy homeostasis function in the pectoral muscles of affected chickens. This observation is in agreement with previous studies which showed mitochondrial damage in affected chickens beginning from the 4^th^ week of age [7], as well as alterations in energy metabolism in affected chickens at market age [14]. Additionally, the expression of the gene *citrate synthase* (*CS*) (log2FC -0.56), considered as an important biomarker of mitochondrial content in skeletal muscles [28] was downregulated in affected chickens, indicating decreased mitochondrial content, and hence, reduced muscle bioenergetics capacity in affected chickens. Indeed, in line with the study by Hakamata *et al*. (2018) who revealed a lower mitochondrial CS activity, and hence, decreased mitochondrial content in pectoral muscles of fast-growing modern broiler chickens [28], it is conceivable that dysregulation of muscle bioenergetics will be exacerbated in Wooden Breast-affected chickens.

Analysis for disease and biological processes showed evidence of vascular disease in affected chickens, inflammatory response, metabolic dysregulation, ECM remodeling and impairment of excitation-contraction coupling. However, there are significant overlapping pathways and biological processes owing to the overlapping roles of associated genes. Consequently, some of these biological pathways and processes would be analyzed together in the context of WBD.

#### Evidence of vascular disease

Analysis of DE genes from pectoral muscles of chickens at week 3 of age revealed evidence of vascular pathology of the arterial end in affected chickens as depicted by arteriosclerosis and atherosclerosis using IPA. Although arteriosclerosis and atherosclerosis conditions in WBD have not been described in histology at market age, arterial-sparing phlebitis has been extensively observed in previous studies [6–8]. Therefore, the current observation suggests that besides the veins, the arteries are also likely affected during the early phase of WBD in chickens. Alternatively, the unique venous lesions that frequently characterize WBD, could be exhibiting molecular signatures similar to those of atherosclerosis and arteriosclerosis. Indeed, Papah *et al*. (2017) reported close resemblance of the histological presentation of the arterial-sparing phlebitis to atherosclerosis [7].

#### Early inflammatory response

Analysis of genes at week 3 showed occurrence of early inflammatory response in the pectoral muscles of affected chickens as evidenced by a number of pathways (see Figs 2 and 3 and S3 Table). Firstly, the complement system was predicted to be activated by IPA in affected chickens with Z-score = 1.89, owing to upregulation of several genes in the complement system namely *complements C3*, *C7*, *C1R*, *C1S*, *C1QA*, *C1QB*, *C1QC* including *complement C3a receptor 1* (*C3AR1)* (see S2 Table) involved in this pathway. The complement system comprises plasma proteins as well as membrane-bound regulators and receptors that interact with cells and mediators of both innate and adaptive immune system [29]. The activation of the complement system has not only been observed to be a prerequisite for an inflammatory reaction, but also as part of the earliest events of an inflammatory reaction [29]. Additionally, complement system activation, which is known to be activated by a variety of insults including aseptic injury, is widely linked with acute inflammation [29]. Therefore, the activation of the complement system in the current study suggests the existence of early inflammatory reaction in affected chickens. Secondly, the present analysis revealed activation of acute phase response system (Z-score = 2.12) in pectoral muscles of affected chickens at week 3 of age. This is evidenced by the upregulation of acute phase proteins such as the chicken transferrin (*TF*) *ovotransferrin* (*ovo*-*TF*), *complement C3* (*C3*), *serpin family member 2* (*SERPINF2*) and *TNF receptor superfamily member 1A* (*TNFRSF1A* (see S2 Table) in affected chickens. It is known that the levels of positive acute phase proteins frequently increase in response to inflammation [30]. Therefore, like the complement system, the activation of acute phase response system in the present study is an indication of initiation of an inflammatory reaction at an earlier age. In support of this observation, the current study revealed upregulation of specific genes highly linked with inflammatory reaction in the pectoral muscles of birds at week 3. These genes include *interleukin2 receptor subunit gamma* (*IL2RG*) also known as *common gamma chain*, and *TNF receptor superfamily member 1A* (*TNFRSF1A*), both of which were upregulated in affected chickens at 3 weeks of age. *IL2RG* gene encodes the cytokine receptor of interleukin-2 (IL-2) receptor subunit gamma (common gamma chain), which is an IL-2 receptor subunit common to several interleukin receptors such as IL-4, IL-7, IL-9, IL-15 and IL-21 [31]. IL2RG plays a key role in the lymphoid development [31], and it is therefore an important component in inflammatory responses. *TNFRSF1A* gene, on the other hand, encodes the soluble or membrane-bound tumor necrosis receptor 1 (TNF-R1), which interacts with tumor necrosis factor (TNF) alpha, an important pro-inflammatory cytokine considered as one of the key mediators of inflammation [32]. Hence, the upregulation of both *IL2RG* and *TNFRSF1A* genes in affected chickens evidenced in the current study supports existence of an early inflammatory reaction in WBD. Additionally, the upregulation of other genes in affected chickens at week 3 associated with inflammatory reaction including *prostaglandin-endoperoxide synthase 2* (*PTGS2*), *colony stimulating factor-1* (*CSF-1*) and its receptor *CSF-1R* serve to augment this observation. Taken together, the transcriptomic assessment of the pectoral muscles at week 3 suggests the occurrence of early inflammatory response in the course Wooden Breast. Previous studies on Wooden Breast reported development of artery-sparing vasculitis in the early phase of disease process [7,8]. Based on this observation, it is likely that the early inflammatory events demonstrated in the present study are directed towards the venous walls and perivascular lipids, causing the phlebitis phenotype reported previously. This observation is also in agreement with the previous study on the disease which demonstrated the initiation of acute inflammatory reaction beginning from week 3 of age as typified by focal infiltration of inflammatory cells into degenerated myofibers [7]. From this time point (3 weeks of age), it is postulated that the scope and intensity of inflammation (vasculitis and myositis) increases as the severity of the disease increases towards market age [7,8].

#### Dysregulation of lipid metabolism

The present study revealed increased expression of genes associated with lipid metabolism, as depicted by activation of clusters in fatty acid metabolism, lipid synthesis and storage in affected birds. Specifically, these clusters contained several genes associated with lipid metabolism that were upregulated in the pectoral muscles of affected chickens. They include *CD36*, also known as *fatty acid translocase*, a transmembrane protein primarily involved in the transportation of free fatty acid (FFA) into the cytoplasm [33]; *fatty acid binding protein 4* (*FABP4*), also referred to as *AP2*, is an intracellular chaperone or an adipokine belonging to the family of intracellular fatty acid binding proteins (FABPs) [34]. FABP4 is involved in binding and intracellular trafficking of hydrophobic molecules such as saturated and unsaturated fatty acids, retinoids, eicosanoids, prostaglandins and fat-soluble vitamins to specific compartments within the cell [34]. Another important gene involved in lipid metabolism which was upregulated in the pectoral muscles of affected individuals is e*xtracellular fatty binding acid protein* (*EX-FABP*) (Log2FC 1.9). *EX-FABP*, also referred to variously as *Ch21* or *p20K*, is a lipocalin involved in selective binding of long chain unsaturated fatty acids [35]. Other lipid-related genes that were expressed in higher levels in affected chickens include *lipase G* (*LIPG*), *lipoprotein lipase* (*LPL*), *perilipin 1* (*PLIN1*), *thyroid hormone responsive* (*THRSP*) also known as *spot14* and *ATP-binding cassette*, *sub-family A*, *member 1* (*ABCA1*) (see S3 Table). Functional analysis of these lipid-metabolism-associated genes in the present study suggests elevated transport, uptake, translocation and consequently deposition and greater availability of lipids across various extracellular and intracellular domains in the P. major muscles during the disease process. This observation agrees with previous studies which demonstrated presence of lipid infiltration in various components of affected P. major muscle at microscopic level, and grossly as lipid-laden white striations on the muscle at market age [6–8]. Similarly, the findings of the current study corroborate the metabolomics evaluations of Wooden Breast at market age, which reported elevated lipids in affected muscles resulting in lipid dysregulation [14]. These observations suggest that lipid metabolism dysregulation frequently associated with Wooden Breast starts early in life and may be linked with the pathogenesis of the disease in chickens.

#### Altered carbohydrate metabolism

This study showed evidence of increased amount of carbohydrates in the pectoral muscles of affected chickens following functional analysis of the DE genes at week 3. Similarly, upstream regulator analysis of the same gene-set demonstrated elevated levels of glucose and fructose in the pectoral muscles of affected chickens at week 3 post-hatch (see S4 Table). In spite of the likely higher levels of carbohydrates (including glucose and fructose) in the P. major muscles of the affected chickens, assessment for the utilization of the respective chemical compounds show evidence of potential alterations and/or shifts from bioenergetic pathways to other metabolic pathways, an observation that was first suggested by Abasht *et al*. [14]. Firstly, the gene expression of *citrate synthase* (*CS*) (Log2FC -0.56), encoding for the enzyme citrate synthase, and *oxoglutarate dehydrogenase* (*OGDH*) (Log2FC -0.65), encoding for alpha-ketoglutarate dehydrogenase complex (KGDHC), both of which are essential enzymes in the mitochondrial tricarboxylic acid (TCA) cycle [36], were downregulated in the pectoral muscles of affected chickens. This suggests a potential reduction of utilization of glucose or fructose in the TCA cycle, and hence, reduced output in the bioenergetic capacity of the P. major muscle. This observation is line with the reduced oxidative phosphorylation pathway, a major canonical pathway detected by IPA as being inhibited in the affected group in the current study. Conversely, the gene *glutamine-fructose-6-phosphate transaminase 2* (*GFPT2*) (log2FC 0.89), which encodes for an enzyme that utilizes glucose/fructose was upregulated in muscles of affected chickens at week 3. *GFPT2* is the first and the rate-limiting step of the hexosamine biosynthesis pathway (HBP) [37,38]. *GFPT2* converts D-fructose-6-phosphate (Fru-6-P) and L-glutamine to L-glutamate and D-glucosamine-6- phosphate (GlcN-6-P), an important precursor of hexosamines such as uridine diphosphate-N-acetyl-D-glucosamine (UDP-GlcNAc) [37]. UDP-GlcNAc, on the other hand, is a substrate for multiple biological processes including biosynthesis of glycans for subsequent glycosylation events, as well as for protein O-GlcNAcylation, i.e. post-translational modification of proteins with O-linked β-N-acetylglucosamine (O-GlcNAc) on their serine and threonine residues [38,39]. Therefore, the upregulation of *GFPT2* indicates an increase in HBP flux in affected chickens, possibly for use in various processes such as biosynthesis of components of the extracellular matrix including proteoglycans, glycoproteins and glycosaminoglycans through glycosylation. Indeed, the gene *mannosyl (alpha-1*,*6)-glycoprotein beta-1*,*6-N-acetyl-glucosaminyltransferase*, *isozyme B* (*MGAT5B*) (Log2FC 0.9), which encodes the enzyme beta (1,6)-N-acetylglucosaminyltransferase involved in the biosynthetic pathway of N-glycans from UDP-GlcNAc [40], was upregulated in the pectoral muscles of affected chickens. Consequently, the upregulation of *MGAT5B* gene suggests enhanced glycosylation events in affected muscle tissue. This finding is further supported by the upregulation of *galectin-1* (*LGALS1*) gene in affected chickens in the current study, whose protein galectin-1, is a glycan-binding protein [41]. Glycosylation has been shown to modify the functions of proteins. For example, positive acute phase proteins such as complement C3 [42] and ovotransferrin [43], whose respective gene expression levels were upregulated in affected chickens in the current study, have been shown to undergo glycosylation thereby modulating their inflammatory responses [42]. This observation, together with enhancement of receptor recognition functions of glycosylation [42], shows a possible link of glycosylation with early inflammatory response in the pathogenesis of WBD in chickens.

Besides glycosylation, the presence of UDP-GlcNAc from HBP in the affected muscles also suggests potential increase of O-GlcNAcylation modification of P. major muscle proteins in the course of Wooden Breast disorder. O-GlcNAcylation modification, which is closely related to phosphorylation events, has been shown to influence the functional properties of proteins. For example, O-GlcNAcylation of diverse contractile and structural proteins of skeletal muscles in rodents, has been shown to modulate their physiology including calcium signaling pathway [44]. Accordingly, disruption of O-GlcNAc homeostasis has been linked with development of many diseases in humans including insulin resistance, diabetes, neurodegeneration and cancer [38,44,45]. Hence, in the present study, it is likely that O-GlcNAcylation impacts the functional properties of P. major muscle potentially contributing to the pathogenesis of WBD. However, the exact role of O-GlcNAcylation in P. major muscles in the pathogenesis of Wooden Breast warrants further investigation.

Taken together, HBP, which subsequently leads to glycosylation and/or O-GlcNAcylation processes in tissues, appears to be one of the important pathways driving the Wooden Breast disease process in chickens. HBP has been shown to be sensitive to changes in nutrient flux, metabolite availability and enzyme activities [39]. It is therefore plausible to hypothesize that the elevated carbohydrate levels demonstrated in the P. major muscles of the affected chickens, activates HBP. This observation agrees with Abasht *et al*. (2016) and Zambonelli *et al*. (2016) who indicated a potential shift of glucose metabolism from glycolysis to HBP in affected chickens at market age [13,14]. This finding further suggests that this alteration starts earlier in the development of WBD than initially suspected. HBP has also been found to be associated with increased endoplasmic reticulum stress, lipid accumulation and inflammation in the liver [46], features that are replicated in the pectoral muscles of affected chickens in current study. Besides HBP, other pathways that were implicated in extensive utilization of glucose in the P. major muscles of affected chickens at market age include sorbitol biosynthesis, glucuronic acid and pentose-phosphate pathways [14].

#### Remodeling of extracellular matrix

Extracellular matrix (ECM) plays a key role in provision of physical framework upon which critical molecular events that necessitate cellular interactions, differentiation, proliferation, migration, growth and survival take place [47]. Consequently, a wide range of physiological responses, and to some extent, pathological changes are coordinated by the ECM [47]. Remodeling of the ECM leading to fibrosis is arguably one of the most conspicuous features of WBD, frequently characterizing the chronic phase of the disorder. Fibrosis in WBD, primarily arising from elevated and persistent deposition of collagen fibers by fibroblasts within the P. major muscles, has been demonstrated both at microscopic [6–9] as well as at gene expression [13,15,16] levels. The earliest histological detection of fibrosis at week 4 in this group of birds [7], and the current identification of the same process at week 3 through transcriptome analysis demonstrates the progression of fibrosis from molecular (at week 3) to cellular changes (at week4 and onwards). Indeed, the current study revealed upregulation of several genes directly involved in ECM remodeling such as *CTGF*, *PDGFRA*, *FN1* and *TNC* in WBD-affected chickens. Genes associated with proliferation and function of fibroblasts, such as *fibroblast activation protein alpha* (*FAP*), *fibroblast growth factor receptor-like 1* (*FGFRL1*) and *fibroblast growth factor binding protein 1* (*FGFBP1*) were upregulated in affected chickens. Additionally, genes related to collagen synthesis such as *collagen type XII alpha 1 chain* (*COL12A1*), *collagen type VIII alpha 1 chain* (*COL8A1*), *collagen type VI alpha 3 chain* (*COL6A3*) and *collagen type XIV alpha 1 chain* (*COL14A1*) (see S2 Table) were upregulated in the pectoral muscles of affected chickens. These molecules may be thought to work in concert in mediation of changes of the ECM with respect to composition, architectural and mechanical disposition, as well as biochemical cues and signaling responses in the course of Wooden Breast disease process. This observation is exemplified following examination of the role of individual genes associated with ECM remodeling. In this case, *CTGF*, a profibrotic cytokine, has been demonstrated to possess both physiological functions as evidenced in tissue developmental processes as cartilage and bones, as well as in the pathogenesis of several biological disorders through enhancement of fibrosis [48]. Similarly, the upregulation of *CTGF* in the affected chickens is thought to be involved in the initiation of fibrosis frequently associated with WBD. *PDGFRA*, a key molecule in the PDGFR signaling pathway, has also been shown to cause increased and aberrant deposition of extracellular matrix in fibrotic muscle diseases in humans [49]. Accordingly, blocking of PDGFR signaling have been shown to reduce fibrogenesis in several fibrotic diseases in humans [50]. In the current study, the upregulation of *PDGFRA* in affected chickens suggests activation of the PDGFR signaling, subsequently enhancing deposition of ECM, which leads to fibrosis. *TNC* has also been implicated in several fibrotic diseases, where it stimulates profibrotic responses in fibroblasts and maintains persistence of fibrosis in tissues [51]. Similarly, the upregulation of *TNC* in affected chickens as evidenced in the current study may be thought to be associated with the initiation and maintenance of fibrosis in the course of WBD in chickens. *FN1* gene, encodes for fibronectin, a major extracellular protein besides collagen, involved in several extracellular signaling pathways and remodeling of the ECM by induction of fibrosis [47,52]. Hence, the upregulation of *FN1* together with other related genes in the current study, demonstrates that the process of ECM remodeling during the development of WBD in chickens begins early in life than previously thought.

#### Impaired excitation-contraction coupling

Functional analysis of DE genes at week 3 showed evidence of alterations of excitation-contraction (EC) coupling in the pectoral muscles of affected chickens, as indicated by inhibition of skeletal muscle contractility (Z-score <-2). This observation is in line with the previous histological study, which showed elevated hypercontracted myofibers in affected chickens at the same age [7]. Optimal calcium homeostasis and metabolism is critical in the maintenance of EC coupling in skeletal muscles [53]. It is therefore, likely that impairments in EC coupling in this study could also be linked to dysregulation of calcium homeostasis and metabolism. Previous studies in WBD have demonstrated aberrations of calcium metabolism in affected chickens at market age [13,15]. Similarly, examination of the genes implicated in muscle contraction in the current study exhibit association with calcium metabolism. *Myotubularin-related protein 14* (*MTMR14*), also known as *muscle-specific inositol phosphatase* (*MIP*), which is downregulated in affected chickens, is a phosphoinositide phosphatase expressed primarily in striated muscles [54]. *MTMR14* is involved in maintenance of calcium homeostasis and regulation of excitation-contraction coupling in skeletal muscles. Further, deficiency of *MTMR* in mice has been shown to result in muscle disease through disruption of calcium metabolism [54] as well as metabolic dysregulation and inflammation [55]. Therefore, the downregulation of *MTMR* in affected chickens in this study may be contributing to impairment of EC coupling through alteration of calcium signaling cascade. Like *MTMR14*, *Sarcalumenin* (*SRL*) is primarily expressed in striated muscle cells, where it functions as a calcium binding protein within the sarcoplasmic reticulum (SR), similar to calsequestrin. *SRL* has also been found to buffer calcium in the SR lumen as well as maintenance of calcium pump proteins [56]. In mice, deficiency of *SRL* was found to alter EC coupling [56]. Similarly, the downregulation of *SRL* in the affected muscles may be thought to cause alteration of calcium metabolism, hence, compromising EC coupling. *Synaptophysin-like 2*, (*SYPL2*), also known as *mitsugumin 29* (*MG29*), is a transmembrane protein expressed in the triad junction of skeletal muscles [57]. *SYPL2* is also involved in EC coupling where its deficiency in mice was reported to result in interference of intracellular calcium homeostasis and muscle fatigability [58]. In the current study, the downregulation of *SYPL2* in the affected muscle is consistent with the disruption of EC coupling and alteration of calcium homeostasis in the pectoral muscles affected chickens. *Syncoilin* (*SYNC*), a gene that was downregulated in the pectoral muscles of affected chickens, is also expressed in the striated muscles [59]. Found at the neuromuscular junction, sarcolemma and Z-lines, and as a member of dystrophin-associated protein complex, *SYNC* is involved in the maintenance of myofiber integrity during EC coupling [59]. Therefore, the downregulation of this gene in affected chickens suggests disruption of muscle integrity, which could play a role in myodegeneration observed in Wooden Breast.

### Role of upstream regulators in the development of WBD

Upstream regulator analysis by IPA predicted activation of several upstream regulators involved in regulation of cell growth, ECM remodeling, induction of fibrosis, vascular injuries, inflammatory response and lipid metabolism. *Spi-1 proto-oncogene* (*SPI1*), also referred to as *hematopoietic transcription factor PU*.*1*, was predicted as one of the activated upstream regulators in affected chickens. *SPI1*, a transcriptional factor, is specifically expressed in cells of the hematopoietic lineage [60]. *SPI1* is primarily involved in the development of myeloid and lymphoid lineages [60], and therefore considered to play crucial roles in the inflammatory and immune cell responses observed during the early phase of WBD in chickens. Indeed, this role is appreciated by the direct interaction of *SPI1* with genes such as *colony stimulating factor-1* (*CSF-1*) and its receptor *CSF-1R* (see Table 7), which are primarily involved in inflammatory/immune cell development and functions. It is therefore, not surprising that *CSF-1* was predicted as one of the upstream regulators in the pectoral muscles at week 3. *CSF-1* and its receptor (*CSF-1R*), both of which were upregulated in the affected birds, are involved in the regulation of macrophage proliferation, differentiation, migration and survival [61]. This phenomenon agrees with the observation of infiltration of inflammatory cells into various components of the pectoral muscles at week 3 as reported previously [7,8]. *CSF-1* is also known to directly induce the expression of *EGR1*, which was upregulated in affected birds in the current dataset. *EGR1* gene is involved in wide cellular processes such as vascular wound response, differentiation, proliferation and fibrosis. As a transcription factor, *EGR1* is involved in the induction of a number of genes observed in the current study. This include *CD44* [62], a multifunctional transmembrane glycoprotein involved in both physiological and pathological processes [63]. In the current study, the upregulation of *CD44* in the P. major muscles of affected chickens at week 3 may be linked to the initiation of myoregeneration [63], which was first reported at the same age in affected chickens [7]. In addition, by interacting with its major ligand hyaluronan, *CD44* possibly plays other roles in the affected chickens such as coordination of inflammatory responses [64] and remodeling of ECM [63].

Another key upstream regulator observed in the dataset was *mitogen-activated protein kinase kinase kinase 1* (*MAP3K1*), a member of the mitogen-activated protein kinase kinase kinase (*MAP3K*), which control MAPKK-MAPK signaling cascades [65]. Equipped with both a kinase domain and homeodomain motifs, and upregulated in affected chickens at week 3, *MAP3K1*, also known as *MEKK1*, is involved in regulation of a wide range of biological responses including wound healing, growth, cellular migration, immune cell differentiation and function, and vascular remodeling [65]. Following activation by external stimuli such as growth factors, cytokines, cellular stresses or ligands for heterotrimeric G protein-coupled receptors (GPCRs), *MAP3K1* interacts with any of its numerous binding partners such as Mapk1 or c-Jun to activate NF-kB or JNK signaling pathways, thereby transducing their downstream effects [65,66]. Similarly, the upregulation of *MAP3K1* in P. major muscles of affected chickens may be linked to initiation of myoregeneration as reported earlier [7], as well as associated pathologies such as inflammatory responses on the vasculature and myofibers observed in the current study.

*CCAAT/ enhancer-binding protein alpha* (*CEBPA*), one of the key upstream regulators and also upregulated (log2FC 1.37) in affected chickens, encodes a protein belonging to the enhancer binding protein family. The proteins in this family are known to function as transcription factors in regulating several biological processes such as differentiation, metabolism, and immune cell differentiation and maturation [67]. Based on IPA analysis, C*EBPA* exhibited direct interactions with the largest number of target molecules in the gene list (see S4 Table), making it an important upstream regulator for the transcriptome associated with development of Wooden Breast at week 3. *CEBPA* has been shown to play a role in inflammatory and immune responses [68]. Similarly, in the current analysis, IPA analysis revealed direct interaction of *CEBPA* with its target genes encoding complement *C3* and *complement C3a receptor 1* (*C3AR1*), as well as inflammatory-related genes namely, *PTGS2*, *TNFRSF1A*, *CSF1* and its receptor *CSF1R* (see Table 7 and S4 Table). Since all the named target genes were upregulated in affected chickens, it is likely that *CEBPA*, as an upstream regulator, plays a mediation role in the initiation of early inflammatory response observed in affected chickens at week 3 of age. *CEBPA* is also known to play important roles in adipogenesis as well as mediation of lipid and glucose metabolism and energy homeostasis, as evidenced in studies involving humans [69] and genetically modified mice [70]. In agreement with these observations, the functional analysis of DE genes in present study showed direct interaction of *CEBPA*, as an upstream transcription regulator with its lipid-related target genes namely, *stearoyl-CoA desaturase* (*SCD*), *ATP citrate lyase* (*ACLY*), *perilipin 1* (*PLIN1*), *lipoprotein lipase* (*LPL*) and *FABP4* (see Table 7 and S4 Table).

## Conclusion

The findings in the current study provide significant insights into the molecular mechanisms driving the early pathogenesis of WBD in broiler chickens. Major molecular changes observed to be associated with onset and early progression of WBD include vascular changes, primarily phlebitis. Vascular perturbations in the pectoral muscles could be considered critical for initiation of pathology owing to their direct impact on drainage of metabolic waste including nitrogenous and acidic compounds and carbon dioxide from tissues. Other key molecular features observed in the affected muscles include early inflammatory responses as evidenced by activation of complement system and acute-phase response signaling possibly directed towards venous walls as well as in response to myodegeneration that sets in early in life. Metabolic dysregulation primarily involving utilization of carbohydrates and lipid metabolism, and remodeling of the extracellular matrix which lays ground for fibrosis, were other significant processes associated with the disease development. This study also showed association of Wooden Breast with changes in the muscle physiology as evidenced by impairment of excitation-contraction coupling potentially compounded with dysregulation of calcium metabolism.

The findings in the current study show that major cellular and molecular perturbations in the face of Wooden Breast are already present by 3 weeks of age, before the disease is even clinically evident by palpation. Therefore, mitigation strategies of the myopathy should be targeted to a time frame at or before 3 weeks post-hatch in the future.

## Supporting information

S1 File Identification of genes associated with skin contamination in pectoral muscle. (PDF)

S1 Table Parameters and criteria applied for selection of biopsy samples to be used in RNA-seq analysis. (XLSX)

S2 Table Comparison of DE genes from cranial portion of P. major muscles of chickens affected with Wooden Breast Disease at week 2 and 3 of age. (XLSX)

S3 Table Disease and functional categories of DE genes from P. major muscles affected with WBD at week 3 of age as determined by IPA. (XLSX)

S4 Table Upstream regulators predicted to be activated by IPA in the pectoral muscles of chickens affected with Wooden Breast Disease at week 3 of age. (XLSX)

S5 Table Upstream regulators predicted to be inhibited by IPA in the pectoral muscles of chickens affected with Wooden Breast Disease at week 3 of age. (XLSX)
