# Supplementary material for: Gene expression profiling of the early pathogenesis of wooden breast disease in commercial broiler chickens using RNA-sequencing
Source: PLoS One. 2018 Dec 5;13(12):e0207346. doi: 10.1371/journal.pone.0207346 (PMC6281187; doi:10.1371/journal.pone.0207346)
Supplement: S1 File — (PDF) [file pone.0207346.s001.pdf]

Identification of genes associated with skin contamination in pectoral muscle

Background

Differentially expressed (DE) genes from the current study (weeks 2, 3 and 4) were scrutinized for potential skin contamination inadvertently obtained following muscle biopsy sampling process prior to functional analysis. This scrutiny was important to ensure that all DE genes analyzed downstream were derived from Pectoralis major muscles (affected and unaffected) and that they were largely free from skin contamination, which could impact downstream analysis. It was therefore necessary that a list of skin-derived genes (potential contaminant genes) were known beforehand so that they could be filtered out from the overall list of DE genes across all 3 time points before downstream functional analysis.

Strategy

To accomplish the above objective, we scrutinized for candidate muscle biopsy samples from all 3 datasets (week 2, 3 and 4) that could potentially be used to generate skin-derived genes. The aim was to come up with 2 groups of samples (those without skin contamination vs those with skin contamination), all belonging to the same disease state (affected or unaffected) to avoid confounding effect of disease. Identification of significant differentially expressed genes between the 2 groups using Cuffdiff v2.2.1 software would then provide an idea of genes that are highly enriched in the skin-contaminated samples vs those that are not contaminated with skin. It follows therefore that genes that are highly enriched in the skin contaminated samples become true skin contaminants if found in the DE gene sets of muscle samples analyzed for disease vs

non-disease within each of the three time points. Consequently, such skin gene contaminants become candidates for removal before functional analysis of the DE genes from muscle.

To be able to identify samples from the current dataset that contained the skin tissue, (that would eventually form one group of samples with skin contamination), we checked for the expression levels (FPKM values) of a few genes known to be primarily expressed by the skin across all samples. The candidate skin genes included keratin-associated genes such as *keratin, type I cytoskeletal 9-like* (*KRT9L*), *keratin, type I cytoskeletal 10* (*KRT10*) and *keratin 15* (*KRT15*). The hypothesis was that a muscle biopsy sample with skin tissue would have the skin-derived gene exhibiting higher expression values (FPKM values) compared to the sample that did not have skin contamination. Additionally, the consistency of expression levels of the above listed skin-derived genes in the candidate samples was considered. The hypothesis was that the expression levels of the skin genes should be consistent in all the muscle samples that contained skin tissue compared to those that did not have skin contamination.

Methodology

Upon application of the above strategy, we were able to determine that the affected samples (n =11) from week 3 dataset (4 unaffected vs 11 affected) provided a higher statistical power with sufficient and balanced biological replicates to run differential expression analysis using Cuffdiff v2.2.1, and identification of relevant DE genes. From the 11 affected samples, we identified 6 non-skin contaminated samples and 5 skin-contaminated samples (see Table 1 S1 file). The two groups were run using Cuffdiff v2.2.1 software to yield differentially expressed genes between them. While muscle biopsy samples at week 2 and 4 showed promise for being candidates for

identification of skin-derived genes, the groupings possessed a lower statistical power compared to the sample group at week 3. For example, at week 2, unaffected samples had (2 non-skin contaminated samples vs 4 skin contaminated samples) while affected group had (7 non-skin contaminated samples vs 3 skin contaminated samples). Similarly, week 4 samples had (5 non-skin contaminated samples vs 1 skin contaminated samples) for unaffected group, while affected group had (8 non-skin contaminated samples vs 2 skin contaminated samples).

Upon generation of DE genes following Cuffdiff analysis of non-skin contaminated vs skin-contaminated samples from week 3, we determined that any gene with log2 FC ≥1 from this geneset was a potential skin contaminant and was subsequently removed from DE genes of all 3 datasets of the 3 time points. Only those genes that did not have potential skin contaminants were used for downstream functional analysis.

Table 1 S1 file. Week 3 affected samples used to identify skin-derived genes

**Non-skin contaminated samples**	**skin-contaminated samples**
1795_wk3_Mod2	446_wk3_Mod1
1737_wk3_Mod3	362_wk3_Mod4
1794_wk3_Mod6	339_wk3_Mod5
458_wk3_Sev3	1651_wk3_Sev1
457_wk3_Sev4	1748_wk3_Sev6
390_wk3_Sev5	
